# Using Artificial Intelligence to Predict Mechanical Ventilation Weaning Success in Patients with Respiratory Failure, Including Those with Acute Respiratory Distress Syndrome

**DOI:** 10.3390/jcm13051505

**Published:** 2024-03-05

**Authors:** Tamar Stivi, Dan Padawer, Noor Dirini, Akiva Nachshon, Baruch M. Batzofin, Stephane Ledot

**Affiliations:** 1 Department of Anesthesia, Critical Care and Pain Medicine, Hadassah Medical Center, Ein Kerem, POB 12000, Jerusalem 9112001, Israel; noord@hadassah.org.il (N.D.); akivan@hadassah.org.il (A.N.); baruchb@hadassah.org.il (B.M.B.); ledot@hadassah.org.il (S.L.); 2 Department of Pulmonary Medicine, Hadassah Medical Center, Ein Kerem, POB 12000, Jerusalem 9112001, Israel; dan@hadassah.org.il; 3 Faculty of Medicine, Hebrew University of Jerusalem, Campus Ein Kerem, Jerusalem 9112102, Israel

**Keywords:** artificial intelligence, machine learning, prediction models, mechanical ventilation weaning, acute respiratory distress syndrome

## Abstract

The management of mechanical ventilation (MV) remains a challenge in intensive care units (ICUs). The digitalization of healthcare and the implementation of artificial intelligence (AI) and machine learning (ML) has significantly influenced medical decision-making capabilities, potentially enhancing patient outcomes. Acute respiratory distress syndrome, an overwhelming inflammatory lung disease, is common in ICUs. Most patients require MV. Prolonged MV is associated with an increased length of stay, morbidity, and mortality. Shortening the MV duration has both clinical and economic benefits and emphasizes the need for better MV weaning management. AI and ML models can assist the physician in weaning patients from MV by providing predictive tools based on big data. Many ML models have been developed in recent years, dealing with this unmet need. Such models provide an important prediction regarding the success of the individual patient’s MV weaning. Some AI models have shown a notable impact on clinical outcomes. However, there are challenges in integrating AI models into clinical practice due to the unfamiliar nature of AI for many physicians and the complexity of some AI models. Our review explores the evolution of weaning methods up to and including AI and ML as weaning aids.

## 1. Introduction

Invasive mechanical ventilation (MV) is required in about 40% of the patients admitted to intensive care units (ICUs) [1,2]. This life-sustaining intervention is indispensable for the support of patients with a diverse range of critical conditions affecting the pulmonary, neurological, neuromuscular, and cardiac systems [3,4]. While MV may be crucial for immediate life preservation, its prolonged use may result in complications such as ventilator-associated pneumonia, vocal cord injury, and tracheomalacia. These complications are relatively common, posing a significant clinical risk and contributing to increased morbidity and mortality in ICU patients [1,5].

While the majority of the intubated mechanically ventilated patients in ICUs undergo easy and uncomplicated weaning from the mechanical ventilator, about 25–30% of them fail to wean, resulting in prolonged ventilation and increased mortality [1,6,7]. The weaning process, a crucial step in MV management, involves the process of liberating the patient from mechanical support and removing the endotracheal tube, commonly known as extubation [2]. This process can involve a substantial portion, up to 40–50%, of the overall duration of MV [7,8,9]. Several risk factors for respiratory weaning failure have been investigated over time, including co-morbidities, acute disease severity, and problems involving physiological systems, such as respiratory pump failure and cardiovascular instability [7,10].

A distinct and notable subgroup among ICU patients consists of individuals experiencing respiratory failure attributed to acute respiratory distress syndrome (ARDS). ARDS is characterized by the acute onset of pulmonary edema, not caused by heart failure, with reduced blood oxygen concentrations caused by direct or indirect lung injury. According to the ‘Berlin Criteria’, ARDS is diagnosed as “the ratio of partial pressure of arterial oxygen to fraction of inspired oxygen [PaO_2_/FiO_2_] less than 300 mmHg, with bilateral infiltrates on chest X-ray, in the absence of left atrial hypertension” [11]. The management of this condition primarily involves providing supportive care, often requiring mechanical ventilation [12].

“Lung protective ventilation” is the standard strategy used when mechanically ventilating patients with ARDS. This strategy involves providing low tidal volumes, elevated positive end-expiratory pressure (PEEP), and low end-inspiratory (plateau) airway pressure while allowing for permissive hypercapnia (elevated arterial CO_2_ partial pressures) and maintaining a low driving pressure (P_PLAT_-PEEP, with P_PLAT_ defined as the plateau pressure after the inspiratory pause). The goal is to not only correct hypoxemia but also to reduce pulmonary pressures and volumes in order to prevent volutrauma (trauma to the lung caused by excessive inspiratory tidal volumes) and atelectrauma (a condition where there is a cyclic collapse and expansion of the alveoli) in the so-called “baby lung” (a term that highlights the concept that in early ARDS, respiratory compliance seems to reflect the condition of normally aerated lung tissue; this suggests that the aerated lung is not “stiff” but instead small) to mitigate ventilator-induced lung injury and to improve prognosis [13,14,15,16]. In the presence of refractory hypoxemia, additional measures such as neuromuscular blockade [17], prone positioning [18], and extracorporeal membrane oxygenation (ECMO) [19] may be considered. These initial strategies play a crucial role in the successful treatment of ARDS patients, contributing to later weaning success and reducing mechanical ventilation duration [13,20].

The heightened susceptibility of patients with ARDS to Ventilator-Associated Events (VAEs) is influenced by multiple factors, including prolonged mechanical ventilation and compromised immunological function. Underlying medical conditions associated with ARDS, such as fluid imbalance and traumatic injuries like rib fractures, pulmonary contusion, and pneumothorax, as well as pulmonary aspiration, can result in a greater likelihood of developing VAEs and pneumonia. This situation can further exacerbate clinical management challenges. Recognizing the complexity of ventilator weaning in ARDS and understanding and adhering to best practices for ventilator weaning are crucial for improving the prognosis of patients with ARDS [20,21].

The timely identification of a patient’s readiness to begin weaning from mechanical ventilation, choosing an effective weaning technique, and accurately predicting which patients are ready to be weaned are crucial for managing this challenging process. The aim is to minimize complications while maximizing the success rates [3,7]. This challenge is even more pronounced in patients with ARDS [20].

While numerous protocols and clinical practices guide the process of weaning from mechanical ventilation, there is relatively little information in the literature about ARDS, particularly about using artificial intelligence (AI)-assisted approaches. In this review, our aim is to examine the available literature, examine specific AI applications, and explore the implications of utilizing AI in guiding the weaning from mechanical ventilation of patients with ARDS.

## 2. Traditional Approaches to Weaning

The process of weaning patients from mechanical ventilation is complex, with multiple stages from the initiation of ventilation to liberation and extubation. Delayed or failed weaning leads to increased complications and mortality [7]. According to the recommendations of the 6th International Consensus Conference, patients can be categorized into three groups based on ease of weaning: simple weaning (successful extubation at the first attempt), difficult weaning (up to three spontaneous breathing trials or 7 days to complete weaning), and prolonged weaning (more than three trials or 7 days until successful weaning). The prognosis for patients in the first group, constituting 69% of those undergoing weaning, is favorable, with ICU and in-hospital mortality rates of 5% and 12%, respectively. The remaining 31% of patients, representing groups 2 and 3, have ICU mortality rates as high as 25% [8,9].

Standard clinical practice involves initiating ventilation in full-support mode using Pressure-Controlled Ventilation or Volume-Controlled Ventilation. Following this, specific clinical criteria must be met before progressing further in the weaning process. The essential clinical parameters include the resolution of, or a significant improvement in, the underlying cause for ventilation and ensuring adequate gas exchange, typically reflected by an arterial oxygen saturation > 90% with FiO_2_ < 0.4 or PaO_2_/FiO_2_ > 200 with PEEP ≤ 5 cmH_2_O. Additional criteria include: the absence of fever, satisfactory neurological and muscular status, stable cardiovascular function, appropriate hemoglobin concentrations, and the correction of metabolic and/or electrolyte disturbances [1,7,9]. Factors predicting extubation failure include but are not limited to excessive secretions, a mechanical ventilation duration exceeding 72 h, and upper airway disorders. Previous unsuccessful weaning attempts should also be taken into account [8].

Once patients meet these clinical criteria, the decision as to the readiness for weaning is typically guided either by the physician’s clinical expertise and experience or a systematic assessment of respiratory weaning criteria based on established protocols [5,22,23]. Various respiratory parameters with diverse sensitivity and specificity, such as Airway Occlusion Pressure (P0.1), Maximal Inspiratory Pressure (MIP), the rapid shallow breathing index (RSBI), CROP (Dynamic Compliance, Respiratory Rate, Oxygenation, and MIP), respiratory rate, and vital capacity have all been proposed as predictors of weaning success or failure [5,7]. Among these parameters, the respiratory frequency/tidal volume ratio (RSBI) has been found to be especially useful [7,20]. The inclusion of P0.1 in conjunction with the RSBI appears to enhance its specificity [7]. However, it is important to note that none of these parameters alone is deemed sufficient for an accurate prediction of successful weaning at the individual patient level [23].

Patients exhibiting positive indications of probable successful weaning according to one or more of the predictive parameters mentioned above often proceed to a spontaneous breathing trial (SBT). This method is currently regarded as the most reliable diagnostic test [1]. It involves allowing the patient to breath spontaneously for 30–120 min, either through a T-piece, which provides supplemental oxygen, connected to the endotracheal tube [7], or while providing minimal respiratory support through the mechanical ventilator using Pressure Support Ventilation (PSV) or Continuous Positive Airway Pressure (CPAP). Successful completion of the SBT, as evidenced by successful extubation, is achieved in some patients. However, approximately 25–40% of patients fail the first SBT, and 10–25% experience extubation failure, which necessitates re-intubation [9,22,23].

It is worth noting that assessing the impact of spontaneous ventilation in ARDS patients during weaning from mechanical ventilation presents a complex scenario. Although spontaneous efforts can contribute to maintaining diaphragmatic strength, consequently lowering the risks linked to diaphragm atrophy and dysfunction, they might also encourage regional variations in stress and strain, potentially playing a part in the advancement of lung injury [20,24]. Initial studies suggest beneficial effects of spontaneous ventilation on hypoxemia improvement and pulmonary compliance, with reductions in both the duration of mechanical ventilation and the length of stay in the ICU [20,25,26]. However, conflicting results from animal studies highlight potential risks of this approach, including increased transpulmonary pressure and respiratory complications. The balance between positive outcomes and potential disadvantages such as diaphragmatic injury and inflammatory response emphasize the need for cautious consideration in patients with ARDS undergoing weaning from mechanical ventilation. Despite the significant interest in this area, conclusive studies on the true impact of spontaneous ventilation during weaning in ARDS patients remain limited [26].

In recent decades, new modes of mechanical ventilation that include advanced closed-loop systems have emerged. These are a useful adjuncts to the clinical/criteria-based approach described above. Notable modes include Adaptive Support Ventilation (ASV, Hamilton Medical, Rhazuns, Switzerland [27]), SmartCare (Dräger, Lübeck, Germany [28]), Neurally Adjusted Ventilatory Assist (NAVA, Getinge, Gothenburg, Sweden [29]), and Proportional Assist Ventilation (PAV, Medtronic, Minneapolis, MN, USA [30]) (Table 1). What sets these innovative ventilation modalities apart is their patient-adaptive nature, improving synchronization between the patient’s effort and the level of support provided by the mechanical ventilator. Additionally, they can function as intelligent devices that aid in the weaning process [31,32,33], reducing support automatically as the patient’s effort improves, essentially making the weaning process automatic, based on mechanical indices and gas exchange parameters such as SpO_2_ and end-tidal CO_2_.

One example is ASV. This mode integrates measurements of respiratory mechanics and utilizes closed-loop pressure control algorithms to sustain a specific target minute ventilation. The ventilator automatically determines the target ventilatory pattern by considering user inputs and respiratory mechanics data from the monitoring system (resistance, compliance, auto-PEEP). ASV is suitable for the initiation, maintenance, and weaning phases of mechanical ventilation [8,34,35]. SmartCare operates on three core principles: maintaining the patient within a respiratory ‘comfort zone’ by adjusting pressure support levels, gradually decreasing pressure support in stable conditions, and conducting automated spontaneous breathing trials with minimal pressure support. The algorithm relies on parameters like respiratory rate, tidal volume, and end-tidal CO_2_ obtained from ventilator monitoring. Automated weaning trials using SmartCare have demonstrated a positive predictive value of 89%, surpassing conventional weaning procedures (77%) and the RSBI (81%) [31].

While ASV and SmartCare automate weaning by transitioning from controlled to assisted ventilation or implementing continuous weaning protocols, PAV and NAVA provide assisted ventilation proportionate to the patient’s effort [31]. A recent systematic review and network meta-analysis examining the impact of various mechanical ventilation modes in critically ill patients revealed no significant differences in the duration of mechanical ventilation, ICU stay, or hospital stay between PSV, NAVA, synchronized intermittent mandatory ventilation, ASV, PAV, and Smartcare/PS. However, in comparison to PSV, each of the mentioned modes enhanced the success rate of ventilator withdrawal, with PAV standing out notably and NAVA demonstrating a reduction in ICU mortality [36]. These findings are in accordance with an earlier meta-analysis, indicating moderate-certainty evidence that PAV enhances weaning success rates, reduces mechanical ventilation duration, and shortens ICU length of stay (LOS) compared to PSV. Additionally, it is worth noting that NAVA appears to improve in-hospital and ICU survival [33]. Conversely, Linton et al. [37] conducted weaning trials in chronic respiratory patients, demonstrating that ASV is a cost-effective mode, leading to reduced requirements for respiratory therapists and intensive care personnel [35].

The automation of mechanical ventilation is gaining prominence due to the previously noted clinical benefits regarding ventilation duration and support in the weaning process for patients in general. Also, the increasing proportion of elderly patients and, consequently, an increase in the number of patients requiring ventilation in the future are several more reasons for automatic ventilation. The forecasted shortage of clinicians and increasing ICU-related costs contribute to the rationale for this system. While managing critically ill patients, especially patients with ARDS, with the challenge of adjusting suitable low tidal volumes and PEEP and oxygen levels and targeting a lower driving pressure, automated ventilation, adjusting breath by breath, offers a safer and more efficient approach. This system has the potential to reduce the morbidity associated with prolonged mechanical ventilation and reduce the costs associated with patients on mechanical ventilation, which represent a major financial burden [8,31,32]. This poses a significant benefit in environments facing constraints in staffing and resources, such as in developing countries, and also during pandemic conditions such as those seen in the recent COVID-19 outbreak.

In recent years, impressive advancements have occurred in the field of AI across various domains. Within this evolving area, the application of AI is undergoing exploration in numerous medical disciplines, including weaning from mechanical ventilation.

## 3. Role of Predictive Modeling in Weaning

The diverse factors influencing the success or failure of the weaning process are extensive, ranging from the patient’s medical history and the initial reason for mechanical ventilation to respiratory predictors, meaning that none of them alone are deemed sufficient for an accurate prediction of weaning success, as previously discussed. Despite the cumulative advancements in the weaning process, including the utilization of sophisticated closed-loop machines, the rate of extubation failure has not changed dramatically over recent years [22].

Delays in evaluating the readiness of the patient for weaning are a common cause of late weaning. As a consequence, patients with prolonged ventilation might experience airway trauma, dysphagia, delirium following extubation, drug dependencies, ventilator-associated pneumonia, diaphragm and muscle wasting, other forms of increased morbidity, and even higher mortality rates [2,23]. On the other hand, a withdrawal of MV that is too rapid may trigger respiratory collapse, impeding the patient’s recovery and exposing them to the associated risks of ventilator-associated pneumonia or other injuries to the lungs induced by the ventilator [3].

Identifying the right time for weaning from mechanical ventilation is essential, given the associated risks and the lack of a standardized protocol. Variability in protocols across institutions reflects uncertainty, highlighting the potential value of an automated or AI-guided prediction model for informed decision making by clinicians [2,22].

Employing personalized parameters for predictive purposes represents a future trend in precision medicine. Machine learning (ML) techniques offer a practical solution for enhancing this predictive approach [38]. AI is a combination of computer science and physiology aimed at enabling computers to mimic human behavior more efficiently and rapidly than a human. Machine learning, as a field of study in AI, refers to the scientific study of the algorithms and statistical models utilized by computer systems to carry out specific tasks [39], often with higher predictive model quality than traditional statistics.

Recent technologies using machine learning have made significant progress in dealing with complex decision-making challenges across various healthcare domains, resulting in its application in acquired immune deficiency syndrome, cancer, diabetes, anemia, schizophrenia, epilepsy, and the administration of anesthesia. In the ICU, the efficacy of early warning systems predicting the likelihood of physiological decline in critically ill patients has shown success. These systems have proven valuable in anticipating the onset of conditions such as ARDS, sepsis, and pneumonia, achieving noteworthy milestones [22,40,41,42,43].

AI or ML techniques offer promising possibilities for enhancing patient outcomes and supporting clinical decision making by analyzing extensive digital information automatically generated in medical settings, particularly in the ICU. This information may include patient characteristics, arterial blood gas readings, respiratory pattern parameters (pressure, volume, flow, etc.) and even graphical data such as waveforms from electrocardiographs or electroencephalographs [1,6,22,44]. This could facilitate the completion of the clinician’s task, enabling the accurate integration of numerous variables to predict the patient’s weaning potential and helping to make informed decisions. For instance, considerations may include discontinuing paralysis, reducing sedation, or determining the need and correct timing for tracheostomy.

Various ML techniques have been employed to predict weaning from mechanical ventilation. These will be further explored in the following section.

## 4. AI/ML in MV Weaning Prediction

### 4.1. Introduction to AI/ML

Modern medical practice generates massive amounts of data. Such data include high-resolution images, biomarkers, continuous physiologic metrics, genome sequencing, and medical records. The analysis of these data exceeds human capabilities and requires automated processes such as AI to deal with this mass of information [45]. AI is defined as a machine’s ability to possess characteristics of intelligent beings such as environmental perception and the ability to solve problems on its own. Machines can gain their intelligence through algorithms, neural networks, or complex program functions.

ML utilizes algorithms to generate a prediction based on finding specific patterns and relations in given dataset. ML can further be divided into supervised learning, in which data labeling is an inert feature, and unsupervised learning, in which algorithms are trained on unlabeled data [46]. Supervised ML models are mainly used for the classification (identify categories/subpopulations) and regression (predicting continuous values) of a new observation based on a training set, while unsupervised ML models are used for clustering (identification of groups within data) and dimensionality reduction (reducing unnecessary data while keeping the principal components of the data) [47].

Deep learning (DL) and neural networks (NNs), in particular, are considered advanced or more sophisticated forms of ML. NNs mimic the nervous system by constructing neural layers such as input, output, and in-between hidden layers. These AI models enable analyses of complex information such as image recognition. DL is characterized by NNs with multiple hidden node layers, making the network sizes larger and allowing the model to be more accurate [47] on the one hand but requiring a larger dataset to train the model [48].

ML models should, on the one hand, provide values or predictions that are close to the training set observations; otherwise, the model is considered to be an underfitted model. On the other hand, ML models should be generalized beyond the scope of the training set or an overfitted model.

The amount of data available will determine the way a supervised ML model will be generated. In cases where there is a sufficient amount of data, the data are divided into three subsets: a training set that will be used to build several models, a validation set that will be used to choose the best-fit model that was built on the training set, and a test set that will evaluate the model’s generalization error. A fully independent test set is considered the gold standard for model validation.

In case the amount of available data is limited, a K-fold cross-validation technique is used. The data are divided into a training set and a test set. The training set is further divided into K subsets, one of which is used for validation and the other, the K-1 subset, is used for training. After K repetitions of this process, wherein in each repetition, a different validation subset is used, the best model is chosen and assessed versus the test set [49].

There are several methods available to generate an ML model. Each method has its own interpretability–performance balance. Each one has its own strengths and weakness. The main methods are listed in Table 2.

### 4.2. AI/ML MV Weaning Models

In recent years, several studies have been performed in order to generate ML/AI-based MV weaning prediction models. These studies used the ML methods described above. Table 3 summarizes the results of these studies.

Many studies used NNs in order to develop an MV weaning prediction model. Hsieh [50] developed an artificial NN (ANN) model using 37 parameters that was trained on data from 3602 ICU patients and had an Area Under the Receiver Operating Curve (AUROC) of 0.85. The same group later developed a 47-feature ANN model that classified the weaning process into categories: simple, prolonged, and difficult weaning. For these categories, this model has AUROCs of 0.910, 0.849, and 0.942, respectively [51].

Kuo [52] developed an ANN for predicting MV weaning based on data from 121 ICU patients that has an AUROC of 0.83 and that was found to be superior to traditional weaning assessment tools such as SBT. Another NN model, designed by Kim [23], used a novel DL model called FT-GAT in order to predict a successful SBT and, eventually, extubation. The AUROC of this model was 0.8, with a similar AUROC being found upon temporal validation.

Menguy et al. [9] used a data-mining process and AI on a prospective database of 108 medical ICU patients in order to find predictors of a successful SBT and weaning from MV for at least 72 h after extubation. In their analysis, cardiovascular parameters (reflected in heart rate variability) had a substantial impact on SBT success in addition to respiratory and systemic parameters (respiratory drive and BMI, respectively). Although the association between heart rate variability and ventilation weaning outcome is established [53], not many AI modals use this parameter in their algorithms.

The support vector machine model developed by Fabreget [54] attempts to predict the likeliness of extubation failure, advising ICU physicians to reconsider their decision to extubate. This model, based on data reflecting the state of the patient 2 h before a planned extubation, showed excellent predictive capabilities, with an AUROC of 98.3%.

Hung [55] developed a real-time AI model for predicting successful extubation using only six ventilator-derived features. This random forest model exhibited a strong predictive performance, with an AUROC of 0.976. This model enables the prediction of MV weaning success every 3 min and is easily applicable in clinical practice in the ICU.

Many studies have used many parameters in order to predict the success of MV weaning using their AI models. The addition of many parameters to AI models can increase the AUROC of the model, as can be seen in the two studies by Hsieh [50,51] mentioned above and in a study by Otaguro [56] that used 57 parameters to generate an AI model that had an AUROC of 0.95. However, the high number of parameters used in these models makes them more difficult to use in clinical practice since not all parameters are easily available.

Chen [6] et al. developed a simplified AI model using only 7 parameters (expiratory minute ventilation, expiratory tidal volume, ventilation rate set, heart rate, peak pressure, pH, and age), reporting an AUROC comparable to a previously built 28-parameter AI model that predicts the success in MV weaning in the coming 24 h among cardiac care unit patients (AUROCs: 0.86 vs. 0.88, respectively).

In addition, Kim and colleagues discovered, during the development of their AI model, that the performance of the model does not increase (in terms of accuracy and AUROC) when more than 21 features are included in the analysis [4].

Jia [2] and partners developed a convolutional NN (CNN) explainable prediction model that can assist clinicians in deciding the feasibility of MV weaning within the next hour. This model incorporates an advanced DL approach in addition to classic AI models (e.g., CNN). This aims to provide physicians with an importance assessment of the relevant clinical factors that can assist them in understanding which treatable factors can lead an individual patient to successful MV weaning.

Another explainable prediction model was designed by Pai et al. [57] using the extreme gradient boosting (XGBoost) algorithm; this model had an AUROC of 0.912. In this model, Pai used 20 parameters, of which 6 of them were considered the most important factors in predicting the success of extubation: the Glasgow Coma Scale, Richmond Agitation-Sedation Scale, urine output, injected fluids, Ppeak, and MAP. These parameters are generally thought to be important when treating critical care patients. The use of explainable AI models can reduce the concerns regarding the use of the ‘black box’ nature of AI.

Liu et al. [22] developed a model that predicts the success and timing of MV weaning in two stages: from intubation to the change in the ventilator mode, and from assist control to support mode and the following stage that includes the weaning itself. Each stage was divided into 11 time frames, and the AI system provides the probability of weaning success in the nearest time frames. The implementation of this system in clinical practice led to a shortening of the MV duration by 21 h and a shortening of ICU LOS by 0.5 days compared to previous data, although the weaning success rates were similar.

Many models are based on single-center data obtained from local medical records. However, some studies used an open-access ICU database in order to developed their AI prediction model. The studies by Jia [2] and Kim [4] described above used the Medical Information Mart for Intensive Care (MIMIC) III and IV databases, respectively, which contain clinical data on thousands of admissions to the Beth Israel Deaconess Medical Center in Boston.

Chen et al. [58] used the MIMIC III database to develop their ML predictive model, but in contrast to other studies, this model was developed in order to predict MV weaning failure and not weaning success. This light gradient boosting machine (LightGBM) model was based on 68 features at the initial stage and narrowed down to 36 features with a negligible difference in AUROC (0.8130 vs. 0.8198) and no impact on the feature importance analysis (duration [hours] of ventilation, PaO_2_, PaCO_2_).

Zhao et al. [59] also developed an ML model that predicts the failure of MV weaning. This CatBoost model is based on MIMIC IV data and includes 19 features. This model has an AUROC of 0.835. Feature importance analysis revealed, as in Chen’s study [58], that MV duration and PSV level were the most important factors for predicting the outcome of extubation. Interestingly, this group applied prospective external validation in addition to the conventional internal validation. The AUROC of the validation cohort was 0.803.

Data from the Dutch Data Warehouse, a multicenter database on COVID-19 ICU patients, was used to developed an MV weaning failure predictive XGBoost model designed especially for these patients. This model has an AUROC 0.7, lower than that reported in previous studies. The low AUROC was attributed to the fact that this database is a multicenter database and the fact that each center has its own treatment and monitoring protocol [60].

In contrast to previously described studies which describe the utility of AI in weaning patients from mechanical ventilation during the acute phase of their disease or after surgery, Liao et al. [44] developed an AI system that predicts the optimal timing for the successful weaning of chronically ventilated patients. In this single-center study from Taiwan, seven AI/ML models were evaluated, and XGBoost was the most effective, with an AUC of 0.868. PS, FiO_2_, T-piece trial, mPaw, PEEP, and Acute Physiology and Chronic Health Evaluation (APACHE) II score were the factors that correlated with successful weaning. Applying this model led to a 3% reduction in re-intubation in the first 120 h following extubation and led to a reduction in the ventilation period of 0.5 days compared to historic data.

Another application of pre-intubation MV weaning AI models is the pre-operative assessment of surgical patients in order to plan their postoperative treatment. Chang [38] and colleagues developed a preoperative risk assessment tool for the prediction of immediate postoperative MV weaning of patients undergoing lung resection surgery using a naïve Bayes classifier algorithm. Applying this algorithm led to more time-efficient preanesthetic consults and improved patient satisfaction scores from these consults compared to previous data. In addition, it was found that the use of sugammadex as a reversal agent had a tremendous impact on successful extubation compared to neostigmine.

Most AI models covered in this review deal with patients already intubated and ventilated or patients that are surgical candidates undergoing preoperative evaluation and consultations. However, the application of AI/MV weaning prediction models before the initiation of MV could assist physicians in their decision-making process. A retrospective study by Kim [4] et al. attempted to address this issue. They developed a voting classifier model that predicts the feasibility of successful weaning up to 14 days post-intubation using pre-intubation data. This model has an AUROC of 0.861. Feature importance analysis revealed that lactate concentration, age, the presence of cerebrovascular disease, and blood urea nitrogen are the most important factors that influence the desired outcome.

**Table 3:** Summary of artificial intelligence/machine learning mechanical ventilation models.

Study	AI/ML Model	Type of Patients/Cases	Number of Participants in Training Phase	Factors That Correlate with Outcome	AUROC of Training Phase	External Validation?	AUROC of Validation Phase	Clinical Use	Model Effect on Clinical Practice
Liao et al. [44]	XGBoost	Chronic ventilation	670	PS, FiO_2_, T-piece trial, mPaw, PEEP, APACHE II score	0.868	No	NA	Yes	A 3% reduction in re-intubation in the first 120 h after extubation. A reduction of 0.5 ventilation days.
Menguy et al. [9]	ZGPD	Medical ICU	108	BMI, heart rate variation, P0.1	83% global performance	No	NA	No	NA
Chang et al. [38]	Naïve Bayes classifier algorithm	Surgical patients (lung resection)	709	Estimated postoperative lung function, exercise load	0.912	No	NA	Yes	More time-efficient preanesthetic consults. Improved patient satisfaction scores.
Chen et al. [6]	LR	Cardiac ICU	1439	Expiratory minute ventilation, expiratory tidal volume, ventilation rate set, heart rate, peak pressure, pH, age	0.86	No	NA	No	NA
Jia et al. [2]	CNN	ICU	2299	Richmond Agitation-Sedation Scale, SBT, FiO_2_, ventilator mode, PIP, PEEP	0.94	No	NA	No	NA
Kim et al. [23]	GF-GAT	ICU	832	NA	0.8	Yes (temporal validation)	0.8	No	NA
Kim et al. [4]	VC	ICU	23,242	Lactate concentration, age, presence of cerebrovascular disease, and BUN	0.861	No	NA	No	NA
Otaguro et al. [56]	LightGBM	ICU	117	Duration of MV, age, PEEP, LDH, APTT, GCS, BUN, A-a gradient, CRP	0.95	No	NA	No	NA
Liu et al. [22]	LightGBM	ICU	5873 in 1st stage 4172 in 2nd stage	First stage—FiO_2_, APACH II score, PEEP, mPaw Second stage—n of SBT, n of suctions	1st stage 0.860 2nd stage 0.923	No	NA	Yes	Shortening of MV in 21 h. Shortening of ICU stay in 0.5 day.
Hsieh et al. [50]	ANN	ICU	3602	Therapeutic intervention scoring system score, chronic hemodialysis, RSBI, heart rate, P/F ratio, MEP	0.85	No	NA	No	NA
Pai et al. [57]	XGBoost	ICU	5940	GCS, RASS, urine output, injected fluids, Ppeak, and MAP	0.921	No	NA	No	NA
Chen et al. [58]	lightGBM	ICU	3636	Duration [hours] of ventilation, PaO_2_, PaCO_2_	0.8198	No	NA	No	NA
Zhao et al. [59]	CatBoost	ICU	16,189	Duration of ventilation, PS level	0.835	Yes	0.803	No	NA
Fleuren et al. [60]	XGBoost	ICU COVID-19 patients	883	FiO_2_, Vt, duration of controlled ventilation, CRP, WBC, PLT, BMI	0.7	No	NA	No	NA
Fabregat et al. [54]	SVM	ICU	697	Δt, GCS, BMI, ROX, and Pplat	0.983	No	NA	No	NA
Kuo et al. [52]	ANN	ICU	121	Mean inspiratory time, mean expiratory time, mean Vt, and mean breathing frequency	NA	Yes	0.83	No	NA
Huang et al. [55]	RF	ICU	233	FiO_2_, Ppeak, PEEP, Pmean, RR, Vt	0.976	No	NA	No	NA
Hsieh et al. [51]	ANN	ICU	3602	Older age, APACHE II, and comorbidities (mainly DM)	0.849–0.942	No	NA	No	NA

ICU—intensive care unit, AUROC—Area Under the Receiver Operating Curve, BMI—body mass index, PS—pressure support, FiO_2_—fraction of inspired oxygen, PEEP—positive end-expiratory pressure, BUN—blood urea nitrogen, P0.1—airway closure pressure, mPaw—mean airway pressure, APACHE II—Acute Physiology and Chronic Health Evaluation, PIP—peak inspiratory pressure, Vt—tidal volume, Ppeak—peak pressure, Pmean—mean pressure, RR—respiratory rate, Pplat—plateau pressure, GCS—Glasgow Coma Scale, ROX—respiratory rate–oxygen index, WBC—white blood count, PLT—platelets, CRP—C-reactive protein, PaCO_2_—arterial partial pressure of carbon dioxide, PaO_2_—arterial partial pressure of oxygen, MV—mechanical ventilation, RF—random forest, XGBoost—extreme gradient boosting, LightGBM—light gradient boosting machine, ANN—artificial neural network, CNN—convolutional neural network, SVM—support vector machine, MEP—maximal expiratory pressure, MAP—mean arterial pressure, SBT—spontaneous breathing trial, LR—logistic regression, GF-GAT—feature tokenizer graph attention network, VC—voting classifier, RASS—Richmond Agitation-Sedation Scale.

## 5. Discussion

In recent years, there has been a significant shift toward the digitalization of medical data. This transformation enables clinicians to rapidly retrieve extensive medical information, creating a basis for deeper exploration. This exploration involves harnessing medical big data to formulate precise prediction models regarding the management of a patient in the ICU. Improved accuracy in predictions translates to more effective and consistent healthcare outcomes, helps reduce clinical uncertainty, and enhances patient safety. In this context, AI emerges as a valuable tool in reducing instances of medical errors and minimizing the need for extensive human resource allocation [38].

AI prediction models contribute to a comprehensive risk assessment by evaluating various factors. When these models exhibit notable accuracy, predictive power, and consistent reproducibility, they empower clinicians to conduct personalized risk assessments. This is achieved by exploring information from prior medical records to offer tailored evaluations for individual patients [22,38].

In our review of AI- and ML-based models, we emphasize the need for a reliable tool to assist the physician in decision making for weaning from mechanical ventilation, particularly in the challenging-to-wean ARDS population. Clinical studies employing ML as a tool have demonstrated promising outcomes, including reduced ML durations and shorter LOSs in critical care units in diverse populations [22,44].

An important aspect to note is our specific focus on the ARDS population in predicting weaning from mechanical ventilation through modeling. Although we made efforts to emphasize the application of these models in ARDS patients, we found a limited amount of studies in the literature focusing on this population.

### 5.1. Limitations of Artificial Intelligence

Despite the great possibilities and benefits, it is essential to address concerns regarding decision-making applications involving AI and ML. First, it may be challenging to incorporate the wide array of individual opinions and beliefs that influence patients’ personal choices, along with cultural differences and diverse perceptions of intensive care interventions.

Additionally, ML models may erroneously interpret false associations as real relationships between events. Human oversight is essential to prevent such misinterpretations.

Furthermore, the quality and resolution of data significantly impact the effectiveness of AI models, and the reliance on natural language in clinical documentation poses challenges for AI algorithms. Variations in software systems, local protocols, and medical practices can influence the wide-ranging data that are used to train ML models, further complicating the performance of AI techniques [48,61].

The methods used in AI can sometimes produce nontransparent results, where predictions by algorithm are made without clear explanations of the underlying reasons. The lack of explanatory power and related potential bias is hard to identify. The complexity of AI algorithms, often referred to as black-box systems, can pose challenges for building the trust of the clinical staff and their understanding of the predictions made by AI systems. Consequently, there is a need for ongoing research to enhance the explainability of AI. Moreover, intensivists anticipate that AI can be utilized to develop a decision support tool that considers the overall condition of the patient, going beyond mere predictions about a single condition [22,61].

Various methods exist to achieve explainability, with two pertinent and complementary methods: feature importance and counterfactual explanations. Feature importance involves identifying the most significant features in a model for making predictions, typically ranking these features by importance. To simplify interpretation, complex ML models often employ feature importance methods to build a more understandable model compared to the original. This method is the most common method for achieving explainability. Counterfactual explanations, which were presented by Wachter et al. [62], are explanations after predictions that help clinicians understand ML outputs. Counterfactual explanations suggest changes in patient features and alternative inputs into the ML model in order to yield a more favorable outcome. Having diverse counterfactuals provides flexibility for clinicians to choose feasible changes that could lead to the desired outcome [2].

It is worth highlighting that even with the suggested solutions mentioned above, which help to clarify the intricacies of the models, and while ML can offer valuable insights, these ML tools should be viewed as adjuncts in decision making. They are not substitutes for a thorough clinical evaluation and the consideration of other relevant parameters by the healthcare provider.

Publications detailing innovative ML methodologies in medical applications often rely on data collected retrospectively. As a consequence, evaluating the direct impact of ML methods on clinical outcomes becomes challenging. This review reveals that only a limited number of studies have validated their ML models on a sperate cohort and assessed the practical benefits of ML in real-world clinical scenarios [3,23,59]. Findings suggest that ML holds promise in addressing crucial clinical challenges related to weaning, including predictions for SBT and extubation failures, blood gas predictions, and adjustments in ventilator settings [3].

Further research is necessary to establish effective validation methods for the performance of these systems.

### 5.2. Ethical Considerations

In addition to address the limitations of the MV weaning AI/ML models described above, it is important to recognize the ethical and moral aspect of AI in medicine, which might limit the implementation of AI in clinical practice. A review by Murphy et al. [63] lists four major ethical concerns related to AI implementation in healthcare:

Privacy and security—concern about the collection and use of personal data without the consent of the patients and the potential for these data to be be hacked or reidentified to a specific person.

Trust in AI applications—trust regarding the safety, security, privacy, and appropriate use of personal data in AI applications by both patients and healthcare providers. Hesitancy and mistrust by physicians to use AI technology due to the difficulty to understand and to explain the ML technology (the black box effect, as mentioned above). In addition, there is a fear of us becoming dependent on AI technology, even if the data are inconclusive.

Accountability and responsibility—the question of responsibility for the errors made in clinical practice arise when AI applications are involved in decision-making processes. The complex nature of AI technology combined with the fact that these models were developed by private companies make it more difficult for physicians to inspect and scrutinize them. Since physicians are responsible for their clinical practice, they bear the responsibility for their decisions, including the use of AI. However, AI application developers also share this responsibility to some extent.

Bias—since AI/ML algorithms were developed by humans, bias is embedded in them and in the data used to train them. Developers tend to reflect their or society’s values in their algorithms, and these values might be different from those of other societies around the globe. There is also a fear of the overrepresentation of one group over other groups. The lack of generalizability can influence the performance of AI models.

It is worth highlighting that while AI offers valuable insights and shows promise in disease classification, patient stratification, and precision medicine, it should be viewed as a supportive tool in decision making. It cannot substitute thorough clinical evaluation and the consideration of other relevant parameters by the healthcare provider. AI also cannot replace the human touch in patient communication [48].

### 5.3. Future Directions

As described in this review, the ability of AI algorithms to sift through vast data and uncover complex correlations makes them essential aids to clinicians. Looking ahead, a notable advancement that could greatly impact the implementation of AI into clinical practice is the potential for upcoming models to feature user-friendly interfaces. This would enable healthcare professionals to effortlessly input data via mobile apps and seamlessly receive recommendations, bypassing the complex mathematical aspects involved in the development of ML models. To effectively integrate and implement AI methods in the clinical realm, collaboration among experts from various disciplines, including computer science, engineering, and healthcare providers, will be essential.

AI/ML models for weaning from mechanical ventilation are in a nascent stage of development. As such, these models have not yet been developed or trained in all possible population groups. Clearly, once a general model for weaning has been developed by AI, it will have to be further trained and validated in individual population groups defined by pathophysiological underlying processes and, indeed, other factors such as nutritional status, genetics, and frailty, amongst others. Additionally, it is imperative to meticulously analyze disparities among diverse populations to ensure precise model application and accurate predictions.

## 6. Conclusions

Assessing the optimal timing for weaning from MV in patients is an important task for ICU practitioners. Although weaning from MV is a commonly performed procedure in the ICU, there is still a need to easily identify patients prone to extubation or weaning failure. The medical literature has identified a variety of physiological parameters that have varying abilities to successfully predict weaning outcomes [9]. In this review, we examined AI and ML models that integrate diverse parameters in order to increase the accuracy of weaning predictions. We found that these methods promise to improve MV management.

Our focus on the ARDS population revealed that there is a dearth of studies on the use of MV and AI models in this group of patients. Therefore, future research should aim at validating these models in clinical settings so that clinicians can make data-informed evidenced-based decisions. The ultimate goals are to improve patient outcomes and reduce healthcare costs.

## Figures and Tables

**Table 1:** Types of new modalities of mechanical ventilation include advanced closed-loop systems.

Proprietary Ventilation Mode	Company	Country of Origin
ASV	Hamilton Medical	Switzerland
SmartCare	Dräger	Germany
NAVA	GETINGE	Sweden
PAV	Medtronic	USA

ASV—Adaptive Support Ventilation, SmartCare, NAVA—Neurally Adjusted Ventilatory Assist, PAV—Proportional Assist Ventilation.

**Table 2:** Common machine learning methods.

Machine Learning Method	Description	Usage	Strengths	Weaknesses
Linear regression	Estimates the linear relationship between dependent and independent variables.	Regression	Simple model to implement and understand.	Outliers can affect the regression. Assumes independence between attributes. Not a complete description of relationships among variables.
Logistic regression	Sigmoid function to assign a probability for an event.	Classification	Simple model. Makes no assumptions about distributions. Measures the predictor’s coefficient size and its direction of association. Interpret model coefficients as indicators of feature importance.	Less suitable for complex situations. Assumption of linearity between the dependent and independent variables. Can only be used to predict discrete functions. Cannot solve non-linear problems because it has a linear decision surface.
Decision trees	A flowchart-like tree structure that splits the training data into subsets based on the values of the attributes until a stopping criterion is met.	Classification and regression	Simple to understand and interpret. Deals with unbalanced data. Variable Selection—can identify the most significant variables and the relation between variables. Handles missing values. Non-parametric nature—keeps the model simple and less prone to significant errors.	Overfitting. Sensitive to small variations and alterations in the input data that can drastically change the structure of the decision tree. Biased learning—without proper parameter tuning, decision trees can create bias if some classes dominate.
Random forest	Combination of many overfitted algorithm-generated deep decision trees outputs in order to deal with the bias and overfitting of a single decision tree.	Classification and regression	Reduced risk of overfitting Flexibility—can handle both regression and classification. Can determine feature importance.	Time-consuming process. Requires more resources. More complex model to interpret.
Boosting	A strong classifier model built by a series of weak classifiers in order to decrease the error. Each weak classifier tries to correct the errors present in the previous classifier. This continues till the training dataset is predicted correctly or the maximum number of models are added. Gradient Boosting—boosting technique that builds a final model from the sum of several weak learning algorithms that were trained on the same dataset (numerical or categorical data). XGBoost (v2.0.3) —a regularized version of the t gradient boosting technique. Outperforms the standard gradient boosting method in speed, and the dataset can contain both numerical and categorical variables.	Classification and regression	Improved Accuracy—reduced risk for bias. Reduce the risk of overfitting—reweighting the inputs that are classified wrongly. Better handling of imbalanced data—focusing more on the data points that are misclassified. Better Interpretability—breaking the model decision process into multiple processes.	Vulnerable to the outliers. Difficult to use boosting algorithms for real-time applications. Computationally expensive for large datasets.
K-nearest neighbors	The algorithm places new, unclassified data near its K-nearest neighbors in a field of labeled data points.	Classification	Interpretable results since it relies on proximity calculations. Simple method.	No learning steps. Does not identify the most relevant features to place new data—influenced by noise. Choosing the right K. Computing and time-consuming.
Neural networks	Model that mimics the complex functions of the human brain—activation of a group of neurons from one neural layer activates other neurons in the next layer until the output layer gives the final interpretation of the model.	Classification	Adaptability—the model can adapt to new situations and learn from data. Pattern recognition—excel in audio and image identification, as well as natural language processing. Parallel processing—can process numerous processes at once, improving computational efficiency. Non-linearity—can use non-linear activation functions in order to model and comprehend complicated data.	Computational intensity—training demands a lot of computing power. Black box Nature—difficult to understand how decisions were made. Overfitting. Large training datasets.
Support vector machines	Algorithm used for linear or nonlinear classification or regression. Algorithms find the maximum separating hyperplane in an N-dimensional space between the different classes available in the target feature.	Classification and regression	Perform well with high-dimensional data. Require less memory and use it effectively. Perform well when there is a large gap between classes.	Long training period— not practical for large datasets. Inability to handle overlapping classes and noise. Poorly performed when the number of features for each data point is greater than the number of training data samples.

## Data Availability

No new data were created or analyzed in this study. Data sharing is not applicable to this article.
